# Community-based provision of direct-acting antiviral therapy for hepatitis C: study protocol and challenges of a randomized controlled trial

**DOI:** 10.1186/s13063-018-2768-3

**Published:** 2018-07-16

**Authors:** A. J. Wade, J. S. Doyle, E. Gane, C. Stedman, B. Draper, D. Iser, S. K. Roberts, W. Kemp, D. Petrie, N. Scott, P. Higgs, P. A. Agius, J. Roney, L. Stothers, A. J. Thompson, M. E. Hellard

**Affiliations:** 1 Disease Elimination Program, Burnet Institute, 85 Commercial Rd, Melbourne, VIC 3004 Australia; 2 Department of Infectious Diseases, The Alfred, Melbourne, VIC Australia; 3 Department of Medicine, University of Melbourne, Melbourne, VIC Australia; 4 School of Population Health and Preventive Medicine, Monash University, Melbourne, VIC Australia; 5 Department of Gastroenterology, St Vincent’s Hospital, Melbourne, VIC Australia; 6 Department of Gastroenterology, The Alfred, Melbourne, VIC Australia; 7 Department of Medicine, Monash University, Melbourne, VIC Australia; 8 Centre for Health Economics, Monash University, Melbourne, VIC Australia; 9 New Zealand Liver Transplant Unit, Auckland City Hospital, Auckland, New Zealand; 10 Department of Gastroenterology, Christchurch Hospital, and University of Otago, Christchurch, New Zealand; 11 Department of Public Health, La Trobe University, Bundoora, VIC Australia; 12 Judith Lumley Centre, La Trobe University, Melbourne, VIC Australia

**Keywords:** Community-based care, Hepatitis C virus, Randomized controlled trial, Protocol

## Abstract

**Background:**

To achieve the World Health Organization hepatitis C virus (HCV) elimination targets, it is essential to increase access to treatment. Direct-acting antiviral (DAA) treatment can be provided in primary healthcare services (PHCS), improving accessibility, and, potentially, retention in care. Here, we describe our protocol for assessing the effectiveness of providing DAAs in PHCS, and the impact on the HCV care cascade. In addition, we reflect on the challenges of conducting a model of care study during a period of unprecedented change in HCV care and treatment.

**Methods:**

Consenting patients with HCV infection attending 13 PHCS in Australia or New Zealand are randomized to receive DAA treatment at the local tertiary institution (standard care arm), or their PHCS (intervention arm). The primary endpoint is the proportion commenced on DAAs and cured. Treatment providers at the PHCS include: hepatology nurses, primary care practitioners, or, in two sites, a specialist physician. All PHCS offer opioid substitution therapy.

**Discussion:**

The Prime Study is the first real-world, randomized, model of care study exploring the impact of community provision of DAA therapy on HCV-treatment uptake and cure. Although the study has faced challenges unique to this period of time characterized by changing treatment and service delivery, the data gained will be of critical importance in shaping health service policy that enables the elimination of HCV.

**Trial registration:**

ClinicalTrials.gov, ID: NCT02555475. Registered on 15 September 2015.

**Electronic supplementary material:**

The online version of this article (10.1186/s13063-018-2768-3) contains supplementary material, which is available to authorized users.

## Background

Globally, approximately 71 million people have hepatitis C virus (HCV) infection, and each year 700,000 people die from HCV-related liver disease [1, 2]. The most common mode of HCV transmission in developed countries and increasingly in developing countries, is shared use of injecting equipment by people who inject drugs (PWID) and, therefore, the burden of disease is borne by current or former PWID [3]. Until 2014, the HCV care cascade demonstrated that few people completed diagnostic tests and received treatment [4, 5]. Treatment was only available from specialists in tertiary hospitals, where the need for a referral, inflexible appointment scheduling and lack of multidisciplinary care functioned as a barrier. Further barriers included: the toxicity and variable efficacy of pegylated interferon (PEG)-based therapy, the stigma experienced in healthcare settings and, historically, the need for a liver biopsy [6].

In the era of PEG-based therapy for HCV, many novel models of care were piloted in an attempt to increase the number of people commencing treatment. Strategies included: diversification of care providers to include specialist nurses, or primary care practitioners who had undertaken special training, expanding modes of interaction beyond face-to-face to include telephone or videoconference-based appointments, and offering care outside of tertiary hospitals in venues such as community drug and alcohol services and prisons [7–10]. The impact of these programs was curtailed by PEG-associated toxicity and the limited efficacy of PEG-based therapy.

The discovery of highly efficacious, well-tolerated direct-acting antiviral (DAA) therapy for HCV has revolutionized the treatment landscape, and has allowed innovative models of care to develop in order to improve the HCV care cascade. Globally, multiple treatment models have been implemented and evaluated in response to local DAA funding and prescribing requirements [11–13]. In Australia, since 1 March 2016 and in New Zealand since 1 October 2016, all primary care practitioners have been encouraged to prescribe DAAs, in consultation with a specialist physician if required [14]. All models are attempting to increase the number of people that are treated and cured, especially in priority populations such as PWID, in order to reduce incidence [15]. Given that treatment access policies around the world are currently highly divergent, it is important to obtain data to determine which models of care are most effective and cost-effective at increasing treatment uptake, with the ultimate aims of reducing transmission and preventing morbidity and mortality from HCV.

Some observational studies have reported improvements in retention in care and treatment uptake with the provision of multidisciplinary care to people who have chronic HCV [13, 16]. Further observational data supports HCV care and treatment in the community, usually in a facility with which the patient is already engaged [8, 10, 12, 17]. One small, randomized study of 21 patients suggested that treatment uptake of directly observed PEG therapy was higher in a community treatment center when compared to a tertiary hospital [18]. There is no study that directly compares the difference in retention in care, uptake of DAA treatment and cure outcome, if care and treatment is provided in the community, compared to at a tertiary hospital service. To investigate the effect of community-based HCV treatment on the care cascade, we have designed the Prime Study – to determine the impact of providing DAA treatment in the community on treatment uptake, and to directly compare tertiary hospital and community cure outcomes.

## Methods

### Study design

The Prime Study is an open-label, non-blinded, randomized controlled, effectiveness and implementation study. We initially aimed to recruit and randomly allocate 380 participants. We selected an effectiveness study design to enable comparison of the impact of community treatment on DAA treatment uptake across the two study arms, and simultaneously a non-inferiority study design to assess the effectiveness of community-based DAA treatment relative to an historical control group of people treated with PEG-based therapy. This study’s hybrid design combines testing effects of a clinical intervention on relevant outcomes whilst observing and gathering information on implementation [19]. The study protocol (version 7, 23 February 2017) follows the Standard Protocol Items: Recommendations for Interventional Trials (SPIRIT) Statement, see Additional file 1 (http://www.spirit-statement.org/). This trial has been registered at ClinicalTrials.gov (ID: NCT02555475).

### Study objectives

The primary objective of the study is:

1. To estimate the proportion of people with HCV attending a primary healthcare service (PHCS) who commence DAA treatment at the PHCS and are cured; as measured by sustained virologic response rates 12 weeks post treatment (SVR12)

Secondary objectives are:

2. To estimate the difference in the proportion of people with HCV infection attending a PHCS *who commence DAA treatment* if they are managed at their PHCS, compared to those who are referred to and managed at a tertiary hospital
3. To estimate the difference in the proportion of people with HCV infection attending a PHCS *who obtain an SVR12* if they are managed at their PHCS, compared to those who are referred to, and managed at, a tertiary hospital
4. To define the cascade of care for patients referred to a community hepatitis nurse for assessment of HCV
5. To estimate the reduction in HCV prevalence and corresponding epidemiological impact if service delivery through PHCS was scaled up nationally
6. To estimate the cost-effectiveness of managing and treating people in a PHCS compared to a tertiary hospital

### Study setting

This study is being conducted in Australia (Melbourne and Geelong) and New Zealand (Auckland and Christchurch). Community hepatitis nurses are employed by tertiary hospitals to work in PHCS in the community, providing an outreach service. All PHCS that the community hepatitis nurses attend provide opioid substitution therapy (OST), and many provide a variety of other services, i.e., needle and syringe distribution, and drug and alcohol counseling. In November 2015, eight PHCS affiliated with three tertiary hospital services in Victoria began recruitment. The Australian pharmaceutical benefits scheme (PBS) listing of DAAs on 1 March 2016 created a significant barrier for recruitment; therefore, the study was expanded, and a further five PHCS affiliated with two tertiary hospital services in New Zealand commenced recruitment in February 2017. The New Zealand Government has funded DAAs for people with a MELD < 15 only for genotype (Gt) 1 HCV, and, therefore, recruitment in New Zealand was limited to patients with Gt 1 infection.

### Study intervention

**Fig. 1:**
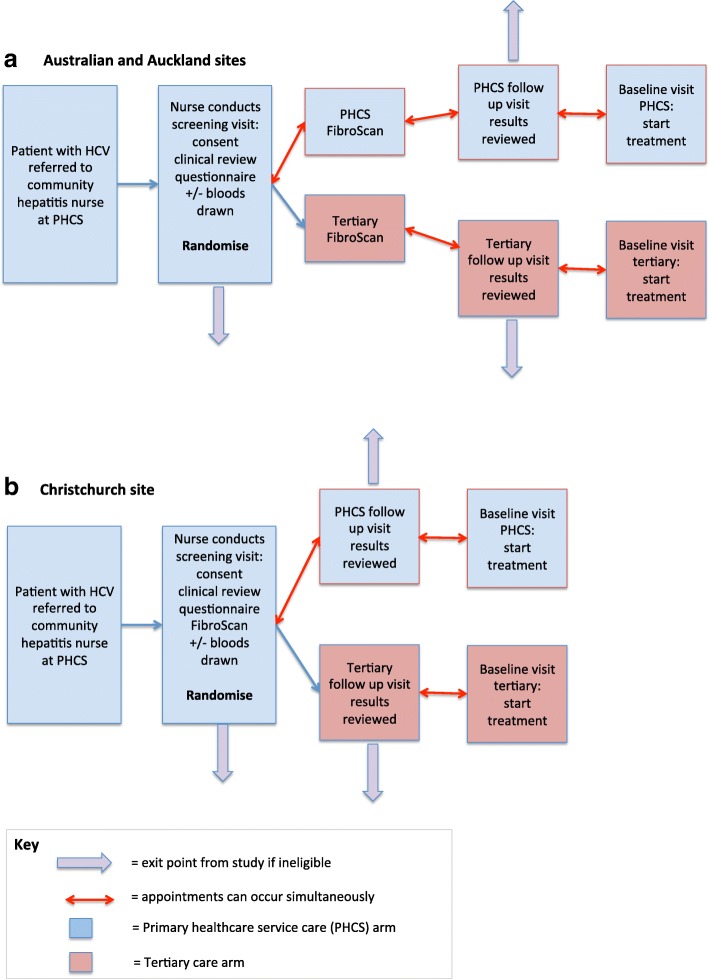
Screening process, eligibility and randomization in the Australian and Auckland sites (**a**) and the Christchurch site (**b**)

After consent has been obtained (see patient informed consent form, Additional file 2), the community hepatitis nurse performs initial screening and participants are randomized into one of two arms, as outlined below (see Fig. 1).

#### Group 1 (standard tertiary care arm)

Participants with chronic HCV infection are referred for 12 weeks of DAAs treatment under the care of specialist physicians (gastroenterologists, hepatologists and infectious diseases physicians) at the local tertiary hospital

#### Group 2 (intervention PHCS arm)

Participants with chronic HCV infection receive 12 weeks of DAA treatment under the care of nursing and medical staff at their PHCS

### Randomization

Although patients are randomized at the end of the screening visit, a unique feature of this study is that randomization occurs before all screening procedures are complete. This is because pre-treatment assessment, which consists of blood tests and transient elastography (TE) (FibroScan**®,** Echosens), is a key part of the care cascade, and given that TE is not readily available in Australia or Auckland, in these study sites the location of TE is determined by the randomization result (see Fig. 1a). In Christchurch, TE is readily available in the community, and participants undergo TE at their PHCS as part of the screening visit (see Fig. 1b).

Participants are randomized to the two study arms using a block randomization approach. Randomization occurs at the PHCS level, to ensure equal numbers of patients from each PHCS are randomized to each arm. Random sequences of permuted blocks are generated, with each block allocating six study participants in a balanced fashion across treatment groups. Randomization is programmed using Stata version 13.0 and provided to study staff in sealed envelopes (Australian sites) or via REDCap database (New Zealand sites).

After the pre-treatment assessment has been completed, the results are reviewed in a follow-up appointment located at the tertiary hospital for group 1, and the PHCS for group 2. Eligible patients are then able to commence DAA treatment.

### Study medications, dosage and administration

The medications being used for this study require a 12-week course of oral therapy.

Participants with genotype 1 are commenced on: paritaprevir 75 mg /ritonavir 50 mg/ombitasvir 12.5 mg co-formulated in one tablet, two tablets in the morning with food and dasabuvir 250 mg one tablet twice daily with food. For genotype 1a weight-based ribavirin was added: ≤ 75 kg 1000 mg daily in two divided doses or ≥ 75 kg 1200 mg daily in two divided doses.

Participants with genotype 3 are commenced on: sofosbuvir 400 mg one tablet daily and daclatasvir 60 mg one tablet orally daily.

Participants with genotype 4 are commenced on: paritaprevir 75 mg /ritonavir 50 mg ombitasvir 12.5 mg co-formulated in one tablet, two tablets in the morning with food and weight-based ribavirin as above.

In Australia, treatment medication is dispensed according to the randomization group. Participants randomized to group 1 fill their scripts at the tertiary hospital pharmacy. Participants randomized to group 2 fill their scripts at a community pharmacy, or have their medications delivered to their PHCS by their community hepatitis nurse. In New Zealand, treatment medication is dispensed according to local practice, which includes some tertiary hospital prescriptions being dispensed from community pharmacies. Participants self-administer the combination therapy.

### Eligibility and recruitment

#### Recruitment

Recruitment is undertaken by the community hepatitis nurses at each study site.

#### Eligibility

Participants must be:

1. Aged over 18 years old; and
2. Able to provide written informed consent; and
3. Have evidence of chronic HCV infection Gt 1^a^, Gt 3, or Gt 4; and
4. Not known to have cirrhosis as defined by; a liver biopsy within 24 months prior to screening demonstrating the absence of cirrhosis, or a screening TE reading < 12.5 kPa^b^, or, if TE is unsuccessful, a screening aspartate aminotransferase to platelet ratio index (APRI) < 1.0 and no clinical or laboratory evidence of cirrhosis; and
5. Be HCV treatment naïve or have previous exposure to PEG / ribavirin therapy only

^a^NZ sites will only recruit Gt 1 participants.

^b^When the study commenced recruitment in November 2015 the initial screening TE was < 9.5 kPa to exclude anyone with advanced fibrosis (METAVIR stages F3–F4). This was increased to < 12.5 kPa on 1 March 2016, to align with the Australian Consensus Statement on HCV treatment that patients without cirrhosis (METAVIR stage F4) can receive community-based treatment [14].

Study subjects are permitted to have elevated alanine aminotransferase (ALT) and AST levels up to 10 times the upper limit of normal, but all of the following parameters have to fall within normal limits as reported by the laboratory: hemoglobin, platelet count, INR, albumin, and direct bilirubin. A creatinine clearance greater than 60 ml/min was required.

Exclusion criteria included (1) known cirrhosis, as defined above, (2) prior exposure to DAA protease inhibitors, (3) HIV or hepatitis B co-infection, (4) hepatocellular carcinoma, (5) pregnancy or breastfeeding, or (6) use of concomitant medication that could not be ceased for the duration of therapy and would result in significant drug-drug interactions with DAAs. In addition, patients who would require ribavirin as part of their therapy were excluded if (1) pre-existing risk factors for anemia were evident, (2) anemia would be medically problematic, or (3) coronary artery disease or cerebrovascular disease had been documented and an acute decrease in hemoglobin up to 4 g/dl as seen with ribavirin would not be tolerated.

### Study assessments

**Fig. 2:**
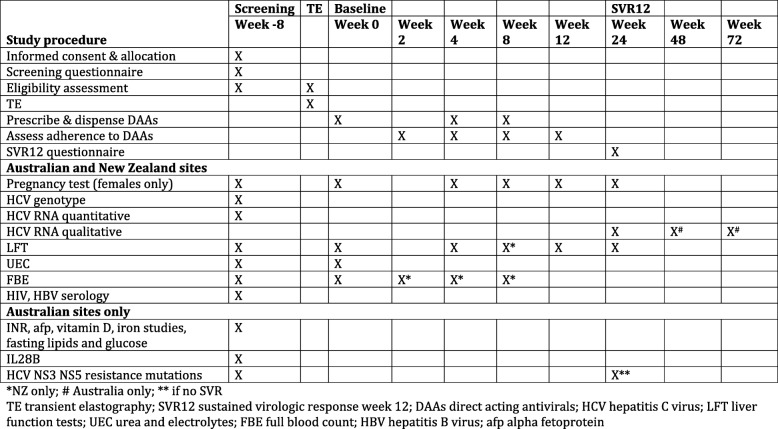
Study schedule

Following randomization, participants complete assessments during the study as outlined in the schedule of assessments (see Fig. 2).

This includes pre-treatment assessment blood tests and TE, and then a clinical review and blood tests at: baseline, on treatment at weeks 2, 4, 8, 12, and post-treatment weeks 12, 24 and 48 weeks, subject to local national guidelines. The visits post treatment at week 24 and 48 are to assess for HCV re-infection. TE is performed pre-treatment and may be incorporated into an appointment, or may be a stand-alone appointment, according to the clinic’s usual practice. Detailed questionnaires are collected at screening and 12 weeks post treatment. Participants are reimbursed AU$20 at the screening visit, and AU$40 at the visit 12 weeks post treatment. Data from assessments is collected on clinical research forms identified only by study number, and entered into REDCap database at Australian sites, or entered directly into REDCap database at New Zealand sites.

### Data sources

At screening and 12 weeks post treatment, the following data is collected:

Demographic characteristics including: age, sex, race and ethnicity, education level, employment status, main income source, average weekly income, living arrangements, type of accommodation and incarceration history.

Drug use including: history of injecting drug use, frequency and substance injected, presence of injecting partners, sharing of injecting drug equipment, receipt of OST, frequency and quantity of alcohol consumed via the AUDIT-C questionnaire.

Health, wellbeing and quality of life indices as measured by the following standardized instruments: the Personal Wellbeing Index, EQ-5D-3 L, 12-item Short Form Health Survey (SF-12) and Patient Health Questionnaire (PHQ-SADS).

At 12 weeks post treatment, customer satisfaction data is collected.

At clinical appointments, the following data is collected: patient-reported adherence to medication, use of a dosette box, administration of medication by a carer or patient, if on OST (and if so frequency of pick-up), AUDIT-C questionnaire regarding alcohol, frequency of injecting drugs and sharing injecting drug equipment, results of pathology tests, any alteration to medication regimen and if the participant has received transport or healthcare worker assistance to attend the appointment.

In addition, data will be collected from the healthcare services participant failure to attend scheduled study appointments.

### Study outcomes

*Primary outcome:*

1. The proportion of people attending a PHCS with chronic HCV who commence DAA therapy at their PHCS, and achieve SVR12

*Secondary outcomes:*

2. The difference in the proportion of participants commencing DAA therapy between group 1 (standard tertiary care arm), and group 2 (intervention PHCS arm)
3. The difference in the proportion of participants achieving SVR12 between group 1 (standard tertiary care arm), and group 2 (intervention PHCS arm)
4. To determine the cascade of care for people with HCV referred to a community hepatitis nurse, measured by the proportion who (1) complete TE, (2) complete blood tests, (3) start treatment (defined as a script being written) and (4) achieve SVR12. Time between diagnosis, assessment and treatment will be determined

### Sample size and power considerations

We designed a non-inferiority study to examine SVR12 outcomes of people treated in a PHCS, and to estimate the effect of providing DAA treatment in PHCS on treatment uptake using intention-to-treat analysis. Prior to the introduction of PEG-free therapy, approximately 10% of people with HCV who attended a PHCS commenced HCV treatment. Of those people, 70% completed therapy, which had an SVR12 rate of 85% [20]. We estimated that the number of people who commenced HCV treatment would substantially increase with DAA therapy to approximately 50% for people managed at PHCS, and 30% for people that attend tertiary hospitals. We attributed most of this difference to loss to follow-up after referral from the PHCS to the tertiary hospital, but before tertiary hospital attendance. We anticipated that 90% would be cured, regardless of whether they are treated at a PHCS or tertiary hospital, and that participants would have high levels of adherence [21].

Based on data from other community cohorts, we estimated 20% of participants would have screening TE results greater than 9.5 kPa, and would be excluded after being randomized. Given these response parameters and equal allocation across study groups, we estimated that 190 study participants were required in each arm in order to address the primary aim of the study and provide sufficient power for secondary aims.

*Aim 1: To determine if SVR12 outcomes of people treated in a PHCS are non-inferior to SVR12 achieved by historic controls.*

In keeping with initial DAA trials, our intervention arm was compared to historical controls [22, 23]. We hypothesized that the rate of SVR12 in people receiving DAA treatment in a PHCS would be non-inferior when compared to an SVR12 rate of 85% for historic controls treated with PEG, ribavirin and simeprevir. A sample of 76 (50% of 152) participants commencing DAA treatment at the PHCS would give a minimum of 90% power to show non-inferiority in those receiving DAA therapy using a 10% margin and an expected SVR12 response of 90% (one-sided α of 2.5%).

*Aim 2: To measure the difference in the proportion of people with HCV infection attending a PHCS who commence DAA treatment, if they are managed at their PHCS, compared to those who are referred to and managed at a tertiary hospital.*

Assuming that 30% of participants in group 1 (standard tertiary care arm) start treatment, our expected sample was powered (80%, 5% significance level) to detect an absolute minimum difference of 16% in treatment initiation between the arms.

*Aim 3: To measure the difference in the proportion of people with HCV infection attending a PHCS who achieve SVR12, if they are managed at their PHCS, compared to those who are referred to and managed at a tertiary hospital.*

Assuming that 27% of participants in group 1 (standard tertiary care arm) achieved SVR12, our expected sample was powered (80%, 5% significance level) to detect an absolute minimum difference of 15.3% between arms.

*Aim 4: To use mathematical modeling, informed by outcomes 1–4, to estimate the reduction in HCV prevalence and corresponding epidemiological impact if service delivery through PHCS was scaled up to a country level.*

We hypothesize that the reduced loss to follow-up and increased treatment uptake when services are delivered through PHCS will result in greater prevalence reduction and reduced incidence compared to projections based on standard tertiary care.

*Aim 5: To use mathematical modeling, informed by outcomes 1–4, to estimate the difference in total healthcare costs, difference in disability-adjusted life years (DALYs), and incremental cost-effectiveness ratio (ICER; AU$ per DALY averted) if service delivery was changed nationally from the standard tertiary care to PHCS delivery.*

This will be based on existing models that account for the costs and DALYs accrued as a result of untreated and progressing liver disease over time [24, 25]. We hypothesize that PHCS delivery will be cheaper than standard tertiary care, and result in fewer DALYs.

### Statistical analyses

For the primary aim (aim 1), an exact one-sided binomial hypothesis test will be undertaken to determine non-inferiority of DAA treatment in PHCS in terms of SVR12 response. For study aims 2 and 3, given the individual-level randomized nature of the design, Pearson chi-square tests [26] of independence will be undertaken in comparing differences in DAA therapy initiation and SVR12 across the two study groups. Statistical analyses will be performed using Stata version 13.0 [27].

### Monitoring

The Prime Study’s Steering Committee meets four times a year to oversee all operational, procedural and policy aspects of the project. The Steering Committee is comprised of gastroenterologists, infectious diseases physicians, senior research fellows, econometricians, a community hepatitis nurse, and a representative from AbbVie. The Committee is responsible for reviewing adverse events, and assessing compliance with the study protocol and with general principles of Good Clinical Practice.

### Amendments to the study protocol

A key challenge for the Prime Study has been to reckon with the highly dynamic HCV treatment environment in Australia. During 2015, when the study was being developed, DAA therapy was not available in Australia outside of clinical trials. Recruitment for the Prime Study initially included only patients with Gt 1, and began in November 2015. Whilst subsidized DAAs were anticipated, it was not known if restrictions would be applied.

In an innovative agreement, the Australian Government negotiated a maximum cap on annual payment to pharmaceutical companies over 5 years, in exchange for providing DAAs, rather than a fixed payment per patient treated, resulting in unrestricted access to DAAs for all Australians living with HCV, effective 1 March 2016. Of note, the PBS listing of DAAs in Australia enabled all medical practitioners (including general practitioners) to prescribe DAAs. The PBS listing of ribavirin-free DAA regimens to treat Gt 1a, including fixed-dose combination sofosbuvir/ledipasvir and sofosbuvir and daclatasvir, significantly impacted on study recruitment. In Australia, two thirds of people with Gt 1 have subtype 1a, and one third have subtype 1b. Recruitment was hampered as study treatment included ribavirin for patients with Gt 1a, whereas ribavirin free DAAs for Gt 1a became available in standard clinical practice. To facilitate recruitment the protocol was amended to include participants with Gt 3 and Gt 4 and the study was expanded to include sites in New Zealand, where paritaprevir/ritonavir/ombitasvir and dasabuvir ± ribavirin is government funded for all patients with Gt 1, whilst access to other DAAs is restricted to patients with a MELD > 15.

In addition, the Australian recommendations for the management of HCV infection, were published on 1 March 2016 [14]. In response, the exclusion criteria were amended to align with the Australian recommendations, changing the TE cut-off from > 9.5 kPa to > 12.5 kPa.

## Discussion

To achieve HCV elimination it is critical to increase treatment uptake, especially amongst PWID. In Melbourne, Victoria the annual HCV treatment rate in PWID prior to the introduction of DAAs was estimated to be three per 1000 [28]. Recent modeling data has suggested that increasing treatment uptake to 59 per 1000 PWID annually could achieve the World Health Organization (WHO) incidence target [24]. For this to occur, treatment must be more accessible to PWID.

Previous studies have demonstrated that PWID can be successfully treated in the community [8], and data from cohort studies indicates that PWID are more likely to undertake treatment if it is offered in the community instead of a tertiary hospital [10]. In Australia, the proportion of individuals receiving their DAA prescriptions from general practitioners in 2016 increased from 4% in March to 19% in September [29]. However, in a recent survey of European patient groups only 20% had access to DAA therapy in non-hospital settings, whilst another European study demonstrated 94% of countries restricted DAA prescribing to specialist physicians only [30, 31].

Data from the US suggests that primary care physician prescription of DAAs is highly effective [32]. To our knowledge, there has been one randomized controlled trial comparing uptake of directly observed PEG-based treatment in the community with standard treatment in a tertiary service [18]. Results of the study supported HCV treatment in the community, but were limited by the small sample size (*N* = 21), and data were not analyzed for statistical significance.

The reason for the paucity of RCT data to inform HCV models of care is that such studies are difficult to perform. As Tait and colleagues recently note in their description of the Tayside, Scotland experience of the transition to multidisciplinary care networks “…. it is very difficult to test the system change and complex interventions undertaken here in a randomised trial, without expensive and large cluster randomised trials, which are probably impossible to perform” [13]. The unprecedented rate of policy change has created a challenging research environment. However, the original study question – does providing HCV treatment in the community increase retention in care, and treatment uptake, without lowering the rate of cure? – remains central to the elimination campaign, nationally and globally.

Whilst conducting a randomized controlled trial of models of care for HCV is challenging in this period of unprecedented change in care and treatment, high-quality evidence is required to shape health services policy that enables the elimination of HCV globally. Data from the Prime Study will be presented mid 2018, and will clarify the role of community-based HCV treatment in the elimination campaign.

## Additional files

Additional file 1: Standard Protocol Items: Recommendations for Interventional Trials (SPIRIT) 2013 Checklist: recommended items to address in a clinical trial protocol and related documents*. (PDF 106 kb)

Additional file 2: Participant information and consent form. (PDF 333 kb)

## Abbreviations

ALT: Alanine aminotransferase
APRI: Aspartate aminotransferase to platelet ratio
AST: Aspartate aminotransferase
DAA(s): Direct-acting antiviral(s)
Gt: Genotype
HCV: Hepatitis C virus
INR: International normalized ratio
MELD: Models for end-stage liver disease
OST: Opioid substitution therapy
PBS: Pharmaceutical benefits scheme
PEG: Pegylated interferon
PHCS: Primary healthcare service
PWID: People who inject drugs
SVR12: Sustained virologic response at week 12
TE: Transient elastography
