# Supplementary material for: Community-based provision of direct-acting antiviral therapy for hepatitis C: study protocol and challenges of a randomized controlled trial
Source: Trials. 2018 Jul 16;19:383. doi: 10.1186/s13063-018-2768-3 (PMC6048874; doi:10.1186/s13063-018-2768-3)
Supplement: Supplementary file 2 — Participant information and consent form. (PDF 333 kb) [file 13063_2018_2768_MOESM2_ESM.pdf]

HREC/15/SVHM/41

Participant Information and Consent Form

**Title**	**Comparing hepatitis C care and treatment in a primary health care service with a tertiary hospital: a randomised trial**
**Short title**	The Prime Study
**Project sponsor**	Burnet Institute
**Principal investigators**	*[Insert Principal Investigator]*
**Location**	

Part 1 What does my participation involve?

1 Introduction

You are invited to take part in research on treatment delivery for Hepatitis C virus (HCV). We have selected you, as you attend a Primary Health Care Service (PHCS) and are infected with genotype 1a or 1b HCV. Most people infected with HCV in Australia do not receive treatment for reasons including concern about the side effects of current government funded medications and location of treatment provider. Therefore we are comparing the outcome of treatment provided in PHCS with treatment provided in specialist hospital clinics.

The medications used to treat HCV in this study are all tablets taken orally, and are called direct acting antivirals (DAA). The DAA tablets are a combination of several types of medications and include paritaprevir/ritonavir/omvitasvir in one tablet and dasabuvir in a second tablet. When packaged together their brand name is Viekira Pak, they work by stopping HCV multiplying in the body.
If you have genotype 1a you will also be prescribed, a third tablet called Ribavirin.

This Participant Information Sheet/Consent Document tells you about the project. It explains the tests and treatments involved if you decide to take part in the project.

Participation in this research is voluntary. If you don’t wish to take part, you don’t have to. You will receive the best possible care whether or not you take part.

If you decide you want to take part in this research project, you will be asked to sign the consent section. By signing it you are telling us that you:

- Understand what you have read;
- Consent to take part in the research project;
- Consent to have the tests, treatments that are described;
- Consent to the use of your personal and health information as described;

2 What is the purpose of this research?

The purpose of this research is to look at the practicality and cost of treating participants in a PHCS compared to a hospital clinic.

The project will investigate if people are more likely to start and complete treatment if it is provided at a service they already attend.

This means that you will be randomised to either receive treatment at the current clinic (PHCS) that you are attending or to attend a hospital clinic. If randomised to attend a hospital clinic you will be referred to a clinic/physician involved in the project by the community nurse.

The HCV treatment for this project (Viekira Pak with ribavirin) is not available for prescription in Australia, although it has been approved by the Australian Therapeutics and Goods Administration. and the Pharmaceutical Benefits Advisory Committee. Viekira Pak with ribavirin is being used in the United States to treat HCV in patients with genotype 1a and 1b. When Viekira Pak is listed on the Pharmaceutical Benefits Scheme, it will be available for prescription in Australia. It is likely that Viekira Pak will be listed on the Pharmaceutical Benefits Scheme by June 2016.

Ribavirin is approved for use in Australia in combination with other HCV medications

This study was initiated by the Burnet Institute and is funded by AbbVie. AbbVie Australia is the pharmaceutical company that produces the study medications

The results of this research may be used by the investigators for the purpose of obtaining a research degree, for example a PhD.

There may be reasons why you are not allowed to take part in this study. The study doctor or staff will discuss these and any other reasons why you may not be allowed to enter the study.

3 What does participation in this research involve?

Medication
Viekira Pak consists of:

- three tablets in the morning
  - Two combination tablets containing; 75mg paritaprevir, 50 mg ritonavir, 12.5mg ombitasvir
  - One 250 mg dasabuvir tablet
- one tablet at night
  - One 250 mg dasabuvir tablet.

Participants with HCV genotype 1a will be prescribed Viekira pak with ribavirin tablets 200mg which will be dispensed separately. Participants with genotype 1b will be prescribed Viekira pak only.
All medications will be in tablet form and need to be taken daily for 12 weeks.
You should never crush or split any of your study pills because they won't work properly. Morning doses of Ribavirin should be taken with food (a meal or snack). You must bring all study medication with you (including empty bottles and packaging) to every visit. You will be given instructions when to return after each visit for your next study visit

Appointments
There are 11 study visits in total:

1. Screening visit; we assess if you are eligible to join the study, request your consent and collect and collect blood tests initial information.
2. Fibroscan and follow up visits; we perform a fibroscan and discuss results.
3. Baseline visit/week 0; treatment is prescribed.
4. Weeks 2, 4, 8 and 12; clinic visits to monitor your health during the treatment period, on weeks 4 and 8 further medication is dispensed.

5. Weeks 24, 36, and 48; are follow-up clinic visits monitoring your health after you finish treatment.

Study Procedure	Screening	Follow up								
**Week**	**- 8**		**0**	**2**	**4**	**8**	**12**	**+12**	**+24**	**+48**
**Medical history**	X	X								
**Concomitant Medications**	X	X	X	X	X	X	X	X	X	X
**Dispense Study Drug**			X		X	X				
**Physical assessment**	X		X		X	X	X	X	X	X
**Fibroscan***	X							X		
**Blood tests**	X		X		X	X	X	X	X	X
**Pregnancy Testing (female participants)****	X		X		X	X	X	X	X	
**Questionnaire**										
**Screening Questionnaire**	X									
**SVR12 Questionnaire**								X		

What will happen during the study visits?

Below we describe what will happen during study visits. If you cannot perform or do not want to go through any of the steps listed below, you should not agree to be in this study.

<u>Medical history and physical examination</u>

The community nurse that you see at the PHCS you attend will ask you questions about your medical history and any medications you are taking to determine if you are eligible for the study. They will also perform a physical assessment for liver disease and to assess your general health. This includes checking your heart rate, blood pressure, temperature and breathing rate, and conducting a urine pregnancy test for women. Pregnancy would exclude participation because ribavirin is dangerous in pregnancy, and the effects of Viekira Pak on pregnancy are unknown. It is a requirement of going on this study that you and your partner agree to use contraception to prevent pregnancy occurring, during the project and for six months after.

At later visits, medical assessments will be done either by the community health nurse or by the doctor at the hospital.

<u>Questionnaire</u>

You will be asked at your first visit (screening) and week 24 to complete questionnaires with reference to;

- Drug and alcohol use
- Injecting behaviours
- Other behaviours that might put you at risk of harm
- Quality of life; health and wellbeing
- Mental health;
- Use of health and social services; and
- Criminal activity

The research involves the collection of information about your use of drugs. That information will be stored in a re- identifiable (or coded) format. In the event that the Burnet Institute is legally required to disclose that information, it may be used against you in legal proceedings or otherwise

The questionnaires will take about 30 minutes to complete.

Blood tests

You will have routine blood tests of less than 20ml of blood taken at 6 clinic visits over the 2 year study period. Blood tests include:

- HCV tests, including measurements of the amount of virus in your blood, the genotype (type) and genes (DNA) of the virus;
- HIV and Hepatitis B virus tests;
- Clinical tests to measure the functioning of your liver, muscles, kidney, blood cells and other body systems, to see how your body is responding to the virus and/or the treatment;

On two occasions (at the beginning and end of treatment) the blood taken for tests will be up to 80ml blood (about 1/3 of a cup) and include;

- Research tests which assess how your immune system responds to the virus;
- Tests of your DNA to see if genetic differences are associated with different responses to HCV disease or treatment (this is not the kind of genetic testing that tells you if you have any genetic disorders that can be passed on through families).

Fibroscan (transient elastography) procedure

A fibroscan test is a non-invasive test like an ultrasound that indicates the level of scarring present in the liver by measuring stiffness. We measure the stiffness using a device that delivers a painless vibration to the skin which then travels through the liver. The speed at which this vibrations returns to the device is recorded as a number. The procedure takes about five minutes and will be performed by the nurse or doctor at your liver clinic. We will repeat the scan 12 weeks after treatment has finished to assess whether there have been any changes in the liver stiffness. Patients found to have higher levels of scarring (cirrhosis) during the first scan will be sent to a specialist hospital clinic for management of their liver disease.

4 What will I be asked to do?

If you take part in the study, you will need to be available to be seen in Melbourne or Geelong for the duration of the project;

5 Other relevant information about the research project

Test results

The blood tests include a screening test for HIV, Hepatitis B virus (HBV) and HCV. You will receive information and counselling before the test. If a test shows you have HIV or HBV, you will have follow-up counselling and medical advice. You will not be eligible to take part in this study if the tests return a positive result for either HIV or HBV. Signing the consent form means that you agree to have this testing; it will not be done without your consent.

However, please note that all positive tests for HIV or HBV must be given to the Victorian Department of Health to help them monitor these diseases; we have to do this under Victorian law.

Data linkage component

The purpose of data linkage is to assess how participants in the Prime Study use health services, and how their use of health services may change over time with their involvement in the study. No information that is collected is used outside of the project and therefore will not affect any information held with any health or public services; such as Centerlink.

We wish to use your personal details so we can record your use of government funded health services. This matching will involve the researchers providing your personal details to other health service agencies, including the Victorian Department of Health (specifically the Victorian Data Linkages unit) and the Australian Government Department of Health and the Department of Human Services (or an accredited linking authority such as the Australian Institute of Health and Welfare).

We will be asking for information from the following sources about specific services provided:

- Medicare Benefits Schedule (MBS): details about your use of health services and the total cost of these services, including doctor's visits, blood and radiology tests;
- Pharmaceuticals Benefits Scheme (PBS): details about medications you have been prescribed and their cost;
- Victorian Admitted Episodes Dataset and Victorian Emergency Minimum Dataset (information about times you have been to a hospital in Victoria).

Only information used to identify you will be sent to other agencies, and the information will be provided in a secure and confidential way. This information may include: Medicare number, first name, surname and middle initial (or a code made up of these); date of birth and gender.

Other information you give us, for example in questionnaires and the outcomes of your blood test will only be seen by the people involved in the study (the research team).

You will be asked to fill out a consent form authorising the study access to your complete Medicare and Pharmaceutical Benefits Scheme (PBS) data as outlined on the back of the consent form. Medicare collects information on your medical visits and procedures, and the associated costs, while the PBS collects information on the prescription medications you have filled at pharmacies. The consent form is sent securely to the Department of Human Services who holds this information confidentially. If you agree to the researchers accessing information about you from Department of Human Services, you will be asked to sign a specific consent form from the Department of Human Services (a separate consent form to the one used for general participation in the study – see below).

Will there be any charge for me to be in this study?
All medication and tests will be provided free of charge to you.

Will I be reimbursed or receive gifts for being in the study?
Participants will be reimbursed $20 at the screening visit and $40 at the SVR 12 visit.

6 Do I have to take part in this research project?

Participation in this research project is voluntary. If you decide to take part and later change your mind, you are free to withdraw from the study at any time. If you consent we will send you a voluntary, questionnaire on why you withdrew from the study.

7 What are the alternatives to participation?

The study nurse will discuss these options with you before you decide whether or not to take part in this research project. You can also discuss the options with your local doctor.

Until the 1st of March 2016, government funded treatment for HCV consists of RBV, pegylated interferon (injections) and for people with genotype 1 simeprevir. On the 1st of March 2016 the following direct acting antiviral drugs will be listed on the Pharmaceutical Benefits Scheme to treat HCV: daclatasvir, sofosbuvir, ribavirin and fixed dose combination sofosbuvir and ledipasvir,

8 What are the possible benefits of taking part?

If the drug works, you may have some from being cured from HCV. If the drug does not work, you may not benefit. Information learned from the study may help other people in the future.

9 What are the possible risks and disadvantages of taking part?

What effects could the tests have on me?

You may feel discomfort during some of these tests or may experience some inconveniences. Some may also have risks, which may include:

- **Blood samples:** drawing blood from your arm may cause pain, bruising, light-headedness, and rarely, infection.

Risks associated with the trial medications
There are risks associated with all medications. The research into Viekira Pak indicates that it is likely to be safer than other HCV treatments. However, there has not been enough research to know this for sure, and Viekira Pak may cause side effects that have not yet been discovered.

The side effects that have occurred with the study medication are listed below. You may have none, some, or all of the effects listed below, and they may be mild, moderate or severe. If you have any of these side effects, or are worried about them, talk with your researcher or study nurse. The nurse will discuss the best way of managing any side effects with you.

Many side effects go away shortly after treatment ends. However, sometimes side effects can be serious, long lasting or permanent. If a serious side effect or reaction occurs, you may be referred to see one of the study doctors and/or advised to stop taking the medication.

Side effects reported in HCV-infected people receiving combinations of the Viekira Pak medicines) and ribavirin (?) in clinical trials of over 2,600 patients included.

- Very common (> 1/10): trouble sleeping (14%), itching (15.7%), nausea (22.3%), tiredness (34.2%), weakness (13.5%), elevated bilirubin (see below), rash
- Common (> 1/100 to < 1/10): low blood count (5.3%).
- Uncommon (> 1/1,000 to < 1/100): ALT elevations (see below)

These events occurred at least 5% more often in patients receiving Viekira Pak medications than with patients who received a sugar pill (placebo).
A small percentage of participants (< 1.3%) permanently stopped treatment with Viekira Pak due to side effects.

Risk of ALT elevations:
About 1% of patients being treated with Viekira Pak medicines experienced an increase in ALT (a substance in blood that increases when your liver is inflamed) levels. Increased levels of ALT happened more often in patients receiving medicines containing ethinyl estradiol, which many birth control pills contain. These increased ALT levels did not cause symptoms, usually happened during the first 4 weeks of treatment, and got better with continued treatment with the Viekira Pak medicines.
Patients who are taking ethinyl estradiol-containing medicines will be asked to stop taking these medicines before starting treatment with the Viekira Pak medicines and advised on other

medications to take during the study. Ethinyl estradiol containing medicines include pills, patches, rings, injections or implants that deliver ethinyl estradiol for the purpose of contraception or for other clinical indications, including some hormone replacement therapy. Whilst you are receiving the Viekira Pak medicines your blood levels of ALT will be measured during this study. Let your doctor know if you have new onset of fatigue, weakness, lack of appetite, nausea and vomiting, jaundice or discoloured faeces.

Increased bilirubin levels

Paritaprevir may temporarily interfere with how bilirubin (a substance produced when red blood cells are broken down by the liver) is handled by your body. 15% patients taking Viekira Pak with ribavirin and 2% of patients taking Viekira Pak had increased levels of bilirubin. Yellowing of the eyes and skin have been noticed in <1% of people. The bilirubin increases are temporary and are not caused by damage to the liver. Increases in bilirubin usually reach their highest level in the first week and get better with continued Viekira Pak treatment.

Ribavirin

Common side effects associated with ribavirin include:

- Haemolytic anaemia (red blood cells are destroyed and removed from the bloodstream before their normal lifespan is over);
- Rash.

Ribavirin may also cause damage to the embryo or unborn child when taken by either parent during or in the 6 months prior to pregnancy.

Some people with certain medical conditions such as ischaemic heart disease, kidney disease or blood conditions may not be able to take the study medications you will have this explained:

Allergic reactions

Any medication has the potential to cause allergic reactions. Common symptoms include rash, hives and itching. If you develop these symptoms, stop taking your study medications and let your researcher or study nurse know as soon as possible.

Serious allergies can cause swelling of the lips, tongue or face, closing of the throat, difficulty breathing, collapse, and rarely death. Seek emergency medical care immediately if you develop any of these symptoms.

Allergic reactions to medicines are rare, and are more likely to occur in people who already have allergies or asthma. If you have asthma, or are allergic to other medications, foods or things in the environment, such as dust or grass, you should tell the doctor or nurse.

Risks associated with pregnancy and breastfeeding

The risks associated with taking Viekira pak while pregnant or breastfeeding are not yet known.

The use of ribavirin during pregnancy is known to increase the risk of birth defects and miscarriage. Ribavirin is also excreted in semen, so there is also a potential risk to the foetus or infant of pregnant or breastfeeding women whose male partners are taking ribavirin.

Low levels of ribavirin can remain in the body for as long as 6 months after the last dose is administered.

Because of the known risks associated with ribavirin, and the unknown risks associated with Viekira Pak, pregnant or breastfeeding women cannot take part in the study.

Due to the increased risk of birth defects and miscarriage associated with ribavirin extreme care must be taken to avoid pregnancy in female patients during the course of treatment and for 6 months afterwards. Men and women that receive treatment must use two forms of contraception, such as the pill and condom – to prevent pregnancy. The study nurse will discuss contraception options with you. Male participants must also agree not to donate sperm to a sperm bank for the purposes of conception from the time you first take your first dose of study medication until 6 months after the last dose of ribavirin.

Risk of deterioration due to HCV
Your condition may or may not improve and could even get worse if you take part in this study. This may be because the treatment you receive is not effective.

The natural course of chronic HCV is that it can slowly damage the liver over many years. 15% of patients infected for 20 years or more will develop severe scarring of the liver, known as cirrhosis.

Viekira Pak +/- ribavirin has been shown to be highly effective in patients that are not cirrhotic.

- Study results have shown a very high cure rate in genotype 1 patients even in patients that have previously received interferon based treatment that has not worked.

These are much better cure rates than existing pegylated interferon based treatments. However, there is still a small (<5%) chance you will not be cured with this therapy.

The nurse/ doctor will monitor your progress during each study visit and look for signs of liver disease. You may be referred for further tests or examinations within a hospital if you become seriously unwell.

Please contact your nurse or doctor if you develop any symptoms that concern you.

Risk of viral resistance

For patients not cured by this treatment, there is a small risk that your HCV may become resistant to Viekira Pak during this study. The risk that your HCV will develop resistance is unknown. It is also not known how long HCV might remain resistant to Viekira Pak. Resistance to Viekira Pak may lead to resistance to other types of anti-HCV drugs similar to the ones taken in this study, which could affect your response to treatment in the future.
Resistant mutations develop most rapidly in people who do not take their full course of medications. Therefore, it is very important to take all your study medication as prescribed by the study staff. If you forget to take some medication, please bring them to your next visit so the research nurse can work out how much you have had in total. Please contact the study staff immediately if you do not feel that you can tolerate the study medication.

Unknown/unexpected risks or adverse events
In addition to the risks listed above, there may be risks that are not known, or very rare; for example, severe reactions to or between the medications, or interactions with other medications.

10 What will happen to my test samples?
Your blood samples will be analysed by the local pathology provider laboratories, and the research storage samples collected for future research will be stored with the Burnet Institute or the Victorian Infectious Diseases Laboratory (VIDRL).

All of your research storage samples will be labelled with your gender and unique study number only. This means only people directly involved in the study will know that your blood samples belong to you.

Your blood samples will be stored indefinitely in locked freezers at the Burnet Institute or VIDRL. After the study is completed, the samples may be used for additional exploratory research, for example, on:

- Pharmacokinetics (how the body processes the medications)
- The structure, function of the virus
- Whether/how the virus develops resistance to the medications
- How the immune system affects the body's response to the virus and medications.

Ethics approval will be required for any additional research studies using your samples.

11 What if new information arises during this research project?

You will be informed of any new information about the disease and treatment by, the nurse or doctor. If you decide to withdraw from the study, the nurse or doctor will discuss options with you regarding ongoing health care.

12 Can I have other treatments during this research project?

Whilst you are participating in this research project, you may not be able to take some or all of the medications or treatments that you normally take.

It is very important to tell the study staff about any treatments or medications you may be taking, including over-the-counter medications, vitamins or herbal remedies, acupuncture or other alternative treatments. You should also tell the nurse or physician about any changes to these during your participation in the research project. Your nurse or doctor should also explain to you which treatments or medications need to be stopped for the time you are involved in the research project.

13 What if I withdraw from this research project?

Your participation in this study is voluntary and you may withdraw from the study at any time by informing a member of the research team or the nurse or doctor. At this point, your participation in the study will end and the study staff will stop collecting information from you. However, the information about you collected prior to the date you withdraw will continue to be used and form part of this study. The Burnet Institute must do this to comply with its legal requirements and to maintain the scientific integrity of the study. Your decision to withdraw from the study or to revoke your authorisation for the collection and use of information about you will not involve any penalty or loss of access to treatment or other benefits to which you are entitled.

Please note that if you withdraw from the project, you will not be able to continue taking the study medication.

If you choose to stop taking the study medication, please tell the study staff so this can be done safely. This will allow the nurse or doctor to discuss any health risks or special requirements. If you choose to stop taking the study medication, you can still remain in the study to continue to provide information. This will be helpful for the purposes of the study.

14 Could this research project be stopped unexpectedly?

You may have to leave the study without your consent if it is considered important for your medical safety or other reasons decided by the researchers. You may be advised to stop taking the trial medication, but remain in the study for monitoring, if:

- You develop serious side effects to the medication;
- You or your female partner becomes pregnant;
- You develop severe complications of liver disease or another serious illness.

15 What happens when the research project ends?

The information collected will be analysed and reported. The results might also be published in scientific journals and/or presented at conferences or seminars. All results will use grouped, de-identified information and you will not be identifiable.

Part 2 How is the research project being conducted?

16 What will happen to information about me?

If you decide to be in this study, the study doctor and research team will use health data about you to conduct this study, as described in this consent form. This may include your name, address, phone number, medical history, photographs, date of birth, and information from your study visits. This health data may come from your family doctor or other health care workers. In addition, information about your participation in this research project may be recorded in your health records at the site and your GP may be informed of your participation.

On-site data collected for this study will be kept *[insert location per institution]* and access will be limited to researchers/staff involved in this study. The data will be stored *[insert length of time of storage]* at *[insert institution].*

By signing the consent form you consent to the research team collecting and using personal information about you for the research project. Any information obtained in connection with this research project that can identify you will remain confidential.

The information collected about you by the Burnet Institute will be stored for 15 years from completion of the research project. Information collected about you from the Commonwealth Department of Human Services will be stored for up to 15 years, and will then be destroyed. Paper and or electronic data forms of the information obtained from the Department of Human Services will be either shredded and/or deleted. To ensure that your personal information is kept confidential, your name and any other information that allows you to be identified directly will be kept separately from all other information collected. Identifying information will not be entered on any study records. Instead, you will only be identified by your initials and a code. The code is used so that the research team can identify you if necessary; for example, to contact you regarding blood results.

All research records will be kept in secure password-protected computer files available only to investigators and study personnel. Paper files will be kept in locked files in locked rooms accessible only to study staff. Your information will only be disclosed with your permission, except as required by law.

By signing the Consent Form, you authorise the release of, or access to, this confidential information to the relevant study personnel and regulatory authorities such as the Australian Government's Therapeutic Goods Administration or as required by law.

The data collected about you from other sources, including the Department of Human Services and Victorian hospitals, will also be linked to that collected in this study. The data describe all the health services you have received from doctors other health practitioners and hospitals, and all the prescription medicines you have received during the specified period of September 2012 until September 2017.

Your data will be stored in the 'Prime Hepatitis Databank', for possible use in future research into HCV and hepatitis medications by the Burnet Institute or collaborators. While future studies using this data will not require further consent from you, they will require specific ethics approval to undertake each study. If Department of Human Services data is being used in future studies, ethics approval will also be sought from the Commonwealth Department of Human Services, and you may be asked to provide additional consent to use this data.

Australian and Victorian laws give you the right to request access to your information collected and stored by the research team. You also have the right to request that any information with which you disagree be corrected. Please contact the study team member named at the end of this document if you would like to access your information.

Information about your participation in this research project will not be recorded in your health records. However, it is recommended that you let your local doctor know about your decision to participate in this research project.

Will information about this trial be included in a Registry Databank?
A description of this clinical trial will be available on http://www.ClinicalTrials.gov. This website will not include information that can identify you. At most, the website will include a summary of the results. You can search this website at any time.

17 Injury

If you suffer an injury as a result of your participation in this research project, please contact the research staff. Hospital care and treatment will be provided by the public health care system (Medicare) at no cost to you if you are eligible for Medicare benefits and elect to be treated as a public patient.

If you think that personal injury has occurred as a result of your involvement in this study, you must contact a member of the research team immediately.

18 Who is funding the research?

This research is funded by AbbVie Australia. AbbVie Australia may benefit financially from this research project; for example, if the project assists in obtaining PBS approval for the medications. However, AbbVie will not have access to the samples and data collected in this project as these will belong to the Burnet Institute.

Other than the reimbursements outlined above, you will not benefit financially from your involvement in this research project even if your samples (or knowledge gained from your samples) prove to be of commercial value. In addition, if knowledge gained through this research leads to discoveries that are of commercial value to AbbVie Australia, the researchers or their institutions, there will be no financial benefit to you or your family from these discoveries.

19 Who has reviewed the research project?

All research in Australia involving humans is reviewed by an independent group of people called a Human Research Ethics Committee (HREC). The ethical aspects of this research project have been approved by the HREC-D of *St Vincent's hospital (Melbourne).* The project will be carried out according to the *National Statement on Ethical Conduct in Human Research (2007)*. This statement has been developed to protect the interests of people who agree to participate in human research studies.

20 Further information and who to contact

The person you may need to contact will depend on the nature of your query. If you want further information about this project or if you have any medical problems which may be related to your

involvement in the project (e.g., any side effects), you can contact the principal doctor on *[phone number]* or a member of the research team, using the specified contact details:

Clinical contact person

**Name**	*[Name]*
**Position**	*[Position]*
**Telephone**	*[Phone number]*
**Email**	*[Email address]*

For matters relating to research at the site at which you are participating or your rights as a study participant, the details of the local site complaints person are:

Complaints contact person

Name	*[Name]*
Position	*[Position]*
Telephone	*[Phone number]*
Email	*[Email address]*

If you have any complaints about any aspect of the project, the way it is being conducted or any questions about being a research participant in general, then you may contact:

Reviewing HREC approving this research and HREC Executive Officer details

**Position**	*Executive Officer of Research*
**Telephone**	*03 9231 3930*
**Email**	*Research.ethics@svhm.org.au*

Consent Document

**Title**	**Comparing hepatitis C care and treatment in a primary health care service with a tertiary hospital: a randomised trial**
**Project number**	
**Project Sponsor**	Burnet Institute
**Principal Investigators**	*[Insert Principal Investigator]*
**Study Nurse**	
**Location**	

Declaration by Participant

I have read the Participant Information Sheet or someone has read it to me in a language that I understand.

I understand the purposes, procedures and risks of the research described in the project.

I give permission for my doctors, other health professionals, hospitals or laboratories outside this hospital to release information to *[Name of Institution]* concerning my disease and treatment for the purposes of this project. I understand that such information will remain confidential.

I agree that the Burnet Institute, study staff, and others may have access to my medical and personal information, as described in this form. I know what will happen to my blood samples collected for this study

I have read this form, and the genetics part of the study has been explained to me in a language I understand

I have had an opportunity to ask questions and I am satisfied with the answers I have received.

I freely agree to participate in this research project as described and understand that I am free to withdraw at any time during the study without affecting my future health care.

I understand that I will be given a signed and dated copy of this document to keep.

Name of participant (please print) ______________________________

Signature ______________________________ Date ______________________________

Declaration by Study Nurse/Senior Researcher[†]

I have given a verbal explanation of the research project, its procedures and risks and I believe that the participant has understood that explanation.

Name of Study Nurse/ Senior Researcher[†] (please print) ______________________________

Signature ______________________________ Date ______________________________

[†]*A senior research team member must provide the explanation of, and information concerning, the research project.*

Note: All parties signing the consent section must date their own signature. I have read this form, and the genetics part of the study has been explained to me in a language I understand

Name of Witness* to Participant's Signature (please print) ______________________________

Signature ______________________________ Date ______________________________

* Witness is not to be the investigator, a member of the study team or their delegate. In the event that an interpreter is used, the interpreter may not act as a witness to the consent process. Witness must be 18 years or older.

PARTICIPANT CONSENT FORM

Consent to release of Medicare and/or Pharmaceutical Benefits Scheme (PBS) claims information for the purposes of **the PRIME Study**

Important Information

Complete this form to request the release of personal Medicare claims information and/or PBS claims information to the PRIME Study.

Any changes to this form must be initialled by the signatory. Incomplete forms may result in the study not being provided with your information.

By signing this form, I acknowledge that I have been fully informed and have been provided with information about this study. I have been given an opportunity to ask questions and understand the possibilities of disclosures of my personal information.

PARTICIPANT DETAILS

1. Mr ☐ Mrs ☐ Miss ☐ Ms ☐ Other ☐

Family name: ____________________ First given name: ____________________

Other given name (s): ____________________

Date of birth: DD/MM/YYYY

2. Medicare card number: ____________________

3. Permanent address: ____________________

Postal address (if different to above): ____________________

AUTHORISATION

4. I authorise the Department of Human Services to provide my:

- ☐ Medicare claims history OR
- ☐ PBS claims history OR
- ☐ Medicare & PBS claims history

for the period* DD/MM/YYYY to: DD/MM/YYYY to the PRIME Study.
*Note: This period cannot exceed 4 ½ years

DECLARATION

I declare that the information on this form is true and correct.

5. Signed: ______________________ (participant's signature) Dated: DD/MM/YYYY

APP 5 – PRIVACY NOTICE

Your personal information is protected by law, including the Privacy Act 1988, and is collected by the Australian Government Department of Human Services. The collection of your personal information by the department is necessary for administering requests for statistical and other data.

Your information may be used by the department or given to other parties for the purposes of research, investigation or where you have agreed or it is required or authorised by law.

You can get more information about the way in which the Department of Human Services will manage your personal information, including our privacy policy at humanservices.gov.au/privacy or by requesting a copy from the department.

A sample of the information that may be included in your Medicare claims history:

Date of service	Item number	Item description	Provider charge	Schedule Fee	Benefit paid	Patient out of pocket	Hospital indicator	Item category
20/04/09	00023	Level B consultation	$38.30	$34.30	$34.30	$4.00	N	1
22/06/09	11700	ECG	$29.50	$29.50	$29.50		N	2

A sample of the information that may be included in your PBS claims history:

Date of supply	PBS item code	Item description	Patient category	Patient contribution	Net Benefit
06/03/09	03133X	Oxazepham Tablet 30 mg	Concessional Ordinary	$5.30	$25.55
04/07/09	03161J	Diazepam Tablet 2 mg	General Ordinary	$30.85	
